# Precision Targeted Ablation of Fine Neurovascular Structures *In Vivo* Using Dual-mode Ultrasound Arrays

**DOI:** 10.1038/s41598-020-66209-0

**Published:** 2020-06-08

**Authors:** Rajagopal N. Aravalli, Dusty Van Helden, Dalong Liu, Parker O’Brien, Hasan Aldiabat, Alexandru-Flaviu Tăbăran, M. Gerard O’Sullivan, H. Brent Clark, John W. Osborn, Emad S. Ebbini

**Affiliations:** 1 Departments of Electrical and Computer Engineering, Minneapolis, MN 55455 USA; 2 Integrative Biology and Physiology, Minneapolis, MN 55455 USA; 3 Comparative Pathology Shared Resource, Masonic Cancer Center and Laboratory Medicine and Pathology, University of Minnesota, Minneapolis, MN 55455 USA; 4 Laboratory Medicine and Pathology, University of Minnesota, Minneapolis, MN 55455 USA; 5 Department of Telecommunications Engineering, Yarmouk University, 21163 Irbid, Jordan; 6 Faculty of Veterinary Medicine, University of Agricultural Science and Veterinary Medicine, Cluj-Napoca, Romania

**Keywords:** Preclinical research, Biomedical engineering, Electrical and electronic engineering

## Abstract

Carotid bodies (CBs) are chemoreceptors that monitor and register changes in the blood, including the levels of oxygen, carbon dioxide, and pH, and regulate breathing. Enhanced activity of CBs was shown to correlate with a significant elevation in the blood pressure of patients with hypertension. CB removal or denervation were previously shown to reduce hypertension. Here we demonstrate the feasibility of a dual-mode ultrasound array (DMUA) system to safely ablate the CB *in vivo* in a spontaneously hypertensive rat (SHR) model of hypertension. DMUA imaging was used for guiding and monitoring focused ultrasound (FUS) energy delivered to the target region. In particular, 3D imaging was used to identify the carotid bifurcation for targeting the CBs. Intermittent, high frame rate imaging during image-guided FUS (IgFUS) delivery was used for monitoring the lesion formation. DMUA imaging provided feedback for closed-loop control (CLC) of the lesion formation process to avoid *overexposure*. The procedure was tolerated well in over 100 SHR and normotensive rats that received unilateral and bilateral treatments. The measured mean arterial pressure (MAP) exhibited measurable deviation from baseline 2–4 weeks post IgFUS treatment. The results suggest that the direct unilateral FUS treatment of the CB might be sufficient to reduce the blood pressure in hypertensive rats and justify further investigation in large animals and eventually in human patients.

## Introduction

Hypertension is a major health care problem worldwide. According to a recent report from the National Health and Nutrition Examination Survey (NHANES), its prevalence among adults > 20 years of age in the United States alone was estimated to be 34% of the total population, which is equivalent to ~85.7 million adults^1,2^. This report projected a significant rise in these numbers in the coming years. Fortuitously, hypertension is treatable; 69 drugs in 15 different classes have been approved for treating hypertension in the US^3^. Despite this, the overall control rate (blood pressure <140/90 mm Hg) is still only 53%^4^, and an estimated 10% to 15% of the general hypertensive population still suffers from drug-resistant hypertension^5^.

It has been well established that excessive activity of the sympathetic nerve system contributes to the development of hypertension, and blood pressure (BP) represents a measurable parameter for this activity^6,7^. The removal of carotid body (CB) has been suggested as a potential therapeutic option for treating chronic systolic heart failure (HF)^8^ and hypertension^9^. CB, located at the bifurcation region of each common carotid artery, is the main peripheral arterial chemoreceptor that senses changes in the levels of oxygen, pH and carbon dioxide in the body^10,11^. A recent study with an animal model of hypertensive HF has shown that CB denervation markedly improved the survival in rats^12^. In fact, single or bilateral removal of CB in clinical studies with >15,000 patients has been reported in the literature with no adverse effects^9^. Therefore, the removal or denervation of CB constitute an effective treatment option for hypertension and HF. In addition to drug treatments, various device-based therapeutic strategies have been implemented to target the CB^13–15^. However, many of these approaches are highly intrusive and require operative surgery.

Image-guided focused ultrasound (IgFUS) is gaining wider acceptance in a wide range of clinical applications as an alternative to surgical resection^16–20^. The feasibility of using FUS to form trackless lesions in biological tissues *in vivo* has been demonstrated for decades^21–24^. Thresholds for thermal coagulative necrosis in terms of the spatial peak temporal average intensity (*I*_SPTA_) for exposure durations in the range of 0.1–100 s have been reported for different tissues. The exposure time range and measured temperature rises due to FUS in a variety of tissues agree well with the thermal dose concept introduced by Sapareto and Dewey^25,26^, thus strongly supporting the thermal damage hypothesis. When the FUS exposure is properly designed, the tissues within the focal zone of the beam undergo coagulative necrosis while the intervening and surrounding tissues are completely spared. Histological evaluation results published by numerous groups worldwide consistently show remarkable demarcation between irreversibly damaged and surrounding healthy tissues^18,27–29^. However, this is mainly true for exposure levels high enough to produce heating rates in the range of 30–60 °C/sec in homogeneous tissues such as the liver. This requires *in situ* intensity levels on the order of 1–8 kW/cm^2^ with durations in the range 1–3 seconds. At these exposure levels, FUS application often produced significant changes in tissue echogenicity indicative of tissue boiling events as was reported in a number of studies^30–32^. Open-loop FUS application at exposure levels higher than the threshold for tissue boiling could result in *overexposure* or excessive damage extending outside the targeted region (see Supplementary Figure 1A). To avoid excessive damage, exposure duration and *in situ* intensity levels were chosen conservatively to produce the desired “cigar shape” and avoid the “tadpole” lesions. This increased the probability of *underexposure*, or failure to form a lesion.

It is now well documented that the tadpole-shaped FUS-induced lesions result from localized tissue boiling^31^. This is typically associated with large changes in echogenicity on real-time ultrasound guidance (e.g. Vaezy *et al*.^33^). The time-to-boiling is determined by the *in situ* intensity, but varies considerably due to tissue heterogeneity, nonlinear propagation, baseline temperature, etc. However, for *in situ* intensities in the range of 5–10 kW/cm^2^, tissue boiling can occur within 1–100 milliseconds.

We have investigated the feasibility of closed-loop control of the lesion formation process using dual-mode ultrasound transducer (DMUT) systems *in vitro* (Casper *et al*.^34,35^, Liu *et al*.^36^). Exquisite control of the lesion size and characteristics was achieved by modulating the FUS intensity upon the detection of the onset of tissue boiling within the target region. The intensity modulation was performed adaptively to adjust the exposure time and intensity based on observed bubble activity at the target.

In the present study, we hypothesized that image-guided, controlled application of focused ultrasound can be used to form precise lesions *in vivo* within the carotid bifurcation region. Furthermore, this can be achieved with minimal collateral damage to surrounding structures, such as the vessel walls and the vagus nerve. The real-time closed-loop control of FUS exposure is enabled by DMUA imaging feedback that guarantees the formation of localized thermal coagulative necrosis in small (~1 mm) targeted tissue volumes *in vivo*.

One of the significant impediments to the widespread acceptance of thermal therapies using high intensity focused ultrasound (HIFU) is the high degree of dependence of the lesion formation process on the local properties of the tissues in the target region^20^. Several groups have recently advocated nonthermal tissue erosion techniques based on more extremely high pressures > 40 MPa with low duty cycles in the range of 0.005–1%^37,38^. These methods, referred to as histotripsy and boiling histotripsy, have the potential for producing more predictable tissue fractionation without inducing thermal damage.

The use of IgFUS for targeting neurovascular structures such as the CBs requires precise application and control of the FUS beam energy in order to produce uniform lesions within the target while minimizing the risk of perforating the nearby vessel(s). The DMUA technology introduced in 2006^39^ and demonstrated *in vivo*^40,41^ is well suited for this application due to the following advantages:

1. The imaging and therapy coordinate systems are inherently registered due to the use of the same transducer elements in forming imaging and therapy beams. Therefore, FUS therapy can be adjusted to target or avoid tissue structures identifiable on real-time DMUA imaging.
2. The DMUA architecture (Casper *et al*., 2013) supports intermittent imaging-therapy operation at frame rates up to 500 fps. It employs a specialized single-transmit focus (STF) imaging for real-time monitoring of lesion formation. STF imaging uses the same beamforming delays for imaging and therapy beams. Thus, STF imaging offers the highest sensitivity exactly within the target voxel, where the FUS-induced tissue modification is most likely to occur.
3. Each STF frame is 1024 × 256 pixels covering a rectilinear grid of 20 mm × 30 mm in the axial and lateral directions, respectively. For the DMUA prototype described herein, the spatial resolution was measured using the speckle cell size method and reported earlier at 0.25 mm and 1.6 mm in the lateral and axial directions, respectively.

These DMUA advantages enabled real-time closed-loop control of FUS exposure with high spatial and temporal precision. The controlled FUS exposure levels produced uniform lesions that are confined to the targeted carotid bifurcation while minimizing the risk of perforation in the nearby blood vessels.

Building on experience from targeting atherosclerotic plaques *in vivo*^42^, we have determined appropriate thresholds for initiating localized boiling events at the FUS focal spot within tens of milliseconds. This was achieved using moderate *in situ* intensity values in the range of 4–10 kW/cm^2^. These boiling events can be detected and localized with high spatial and temporal resolutions as feedback for closed-loop control of the beam intensity to avoid *overexposure* and excessive damage to the surrounding tissues. In particular, a key goal was to minimize or eliminate the probability of perforation. Upon detection of the tissue boiling event, the HIFU intensity is modulated to maintain the boiling within a specified distance from the target point for sufficient duration to guarantee uniform thermal coagulation within the target.

While the DMUA approach has been described previously for IgFUS applications, including *in vivo*^39,41,42^, this the first report of real-time closed-loop control of HIFU for reliably producing uniform thermal coagulation within the carotid bifurcation of the *in vivo* spontaneously hypertensive rat (SHR) model without perforation. The objectives of this study were: (i) testing the feasibility and safety of inducing localized lesions with uniform thermal coagulative necrosis within the carotid bifurcation in spontaneously hypertensive and normotensive rats, (ii) monitoring the changes in the BP, heart rate (HR), body temperature, and breathing rates in animals prior to and after creating the thermal lesions to assess the CB-targeting efficiency of the DMUA system, and (iii) registering histopathological changes that occur at the CB region following the DMUA treatment.

## Methods

### Animals

In this study, 9-wks to 12-wks-old male spontaneously hypertensive rats (SHR) and normotensive Wistar-Kyoto (WKY) rats (Charles River Laboratories, Wilmington, MA), both established from the same parental Wistar stock^43^ were used as treatment subjects and positive controls, respectively (Supplementary Table 1). Animals were housed in a temperature-controlled room with a 12:12-h light-dark cycle. They were fed with normal rat chow (Lab Diet 5012) and distilled water ad libitum. All surgical procedures of this study were approved by the University of Minnesota Institutional Animal Care and Use Committee, and all experiments were performed in accordance with relevant guidelines and regulations.

### Surgical procedures

To continuously measure mean arterial pressure (MAP) and heart rate (HR), a telemetry transmitter (model HD-S10, Data Sciences International (DSI), St. Paul, MN) was implanted into the descending aorta as described previously^44^. Briefly, rats were anesthetized with 5% isoflurane (Piramal Healthcare Limited, Andhra Pradesh, India) and maintained at 2–3% isoflurane for the duration of the surgery. They were then given atropine sulfate (0.5 mg/kg ip; Baxter, Deerfield, IL), gentamicin sulfate (2.5 mg/kg; Hospira, Lake Forest, IL), and meloxicam (2 mg/kg) prior to the surgery. For the telemetry device, a 1-inch midline abdominal incision was made to insert the telemetric transmitter. Another small (1 cm) incision was made in the left groin near the femoral artery to insert the catheter of the transmitter, which was advanced 3 cm into the femoral artery and sutured into place. The body of the transmitter was placed intra-abdominally and sutured to the peritoneal surface. The femoral incision was sutured closed, and the abdominal incision was closed with 9-mm surgical wound clips. Each rat was caged individually. For all surgeries performed, amoxicillin (1 mg/ml in drinking water; West-Ward Pharmaceutical, Eatontown, NJ) and the analgesic Meloxicam (2 mg/kg, daily) were administered postoperatively for 3 days. Animals were allowed to recover from surgery for at least 5 days before beginning the treatment with the DMUA system.

### Experimental setup

Prior to the ablation treatment using IgFUS, animals were anesthetized using isoflurane (Piramal Healthcare Limited; 100% O_2_, 0–5%) inhalation. They were then placed on a platform and the array was positioned on the area covering the carotid region for imaging and FUS treatment with the DMUA. All vital signs were monitored and recorded throughout the procedure.

### Dual-mode ultrasound array system

**Figure 1:**
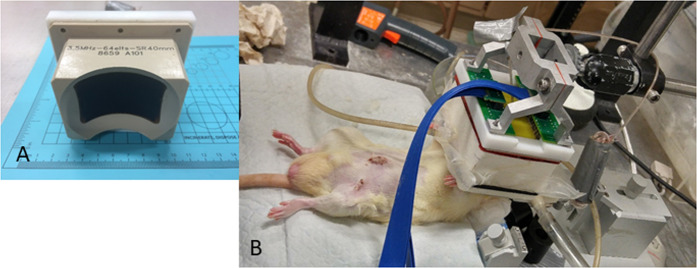
Experimental setup. (**A**) DMUA containing 64-elements (please expand). (**B**) DMUA is placed over the bifurcation region. A water bolus filled with degassed water provides acoustic coupling of the transducer to the skin of the rat.

A concave, 64-element, 3.5-MHz DMUA transducer with 40-mm radius of curvature (Imasonic, Voray sur l’Ognon, France) was used (Fig. 1). The DMUA transducer has a focusing intensity gain of ~4300 at its geometric focus in water with a focal spot volume of 0.4 × 0.6 × 2 mm^3^ in the lateral, elevation and axial directions, respectively. The transducer was described by Haritonava *et al*.^45^ in the context of image-guided application of transcranial FUS *in vivo*. It is capable of imaging in conventional synthetic aperture (SA) imaging mode for guidance and single transmit focus (STF) imaging mode for monitoring. SA imaging at 23 frames/s (fps) provided a sufficiently large field of view (FoV) to visualize important markers such as the trachea and pulsating vessels before and after bifurcation.

Real-time closed-loop control was achieved using an advanced software/hardware architecture described previously^36^. Supplementary Figure 2 shows the system level design of the DMUA system (configured in close loop control mode in this specific case). The system features a custom-built front-end hardware system consists of 32-channel arbitrary waveform transmitter with advanced beam sequencing control and 32-channel receiver and diplexer. Digitized data from transducer elements are streamed to front-end software, where it is beamformed by the GPU beamformer. The GPU beamformer is capable of beamforming synthetic aperture (SA) images at 30 fps and single transmit focusing (STF) in excess of 500 fps in dual mode (or 1000 fps in imaging-only mode). The bubble activity monitoring was performed with STF imaging for its high frame rate and high specificity at focus.

### Image guidance

Real-time 2D SA imaging was used to acquire 3D data sets for identification of the carotid bifurcation. As shown in Fig. 1b), the transducer was attached to a 3-axis mechanical positioner, which could be moved synchronously with real-time 2D SA imaging (at 23 fps). A typical 3D scan was performed by uniformly moving the DMUA in the posterior-anterior while 2D SA frames were acquired starting from a position where the common CA (CCA) pulsation was clearly identified. The motor speed was sufficiently low (typically 0.5 mm/s) to allow the reliable identification of vessel pulsation.

### Monitoring and feedback

STF imaging is a specialized high frame rate mode for real-time monitoring the various bioeffects such as temperature change (500 fps)^45^ and tissue boiling (1000 fps)^30^. It is akin to the popular plane wave imaging used in conjunction with shear wave generation by FUS^46^. Briefly, the same beamforming delays used for the therapy beam are used for the imaging beam with short duration pulses suitable for imaging and at exposures consistent with diagnostic ultrasound. A single, focused transmit beam is used to acquire a frame with dynamic focusing used on receive to form a full frame. In a homogeneous phantom, STF provides an image of the transmit beam. STF imaging achieves the highest sensitivity to echo changes at the target when compared with other guidance systems^30^. At the same time, it provides high sensitivity to any changes induced by the therapeutic FUS beam proximal and distal to the (target) focal spot.

### Closed-loop feedback control of lesion formation

A typical IgFUS lesion formation was performed as follows:

1. Reference SA frames were collected prior to lesion formation.
2. Pre-therapy STF frames (400 fps) were collected for 0.5 s before initiating HIFU and continued for a specified interval (4–10 s), including during HIFU. Integrated backscatter (IBS) from 8 sub-blocks (0.25 mm axially X 1 mm laterally) spanning the HIFU focal spot was computed from each STF frame and baseline values are established. The IBS was calculated as the sum of the pixel intensity values (from beamformed RF data) within each sub-block in every frame.
3. HIFU was switched on at initial *in situ* intensity value suitable for initiating tissue boiling at the focus within 20–40 ms. HIFU and STF transmissions were intermittently applied (Fig. 2) where each HIFU burst was of 1.25 ms duration and each imaging pulse was 1 cycle (approximately 0.31 µs duration), both at 3.2 MHz carrier frequency. The HIFU bursts during this initial state are referred to as the (boiling) *initiation bursts*. Typical intensity values for the initiation bursts was ~7.2 kW/cm^2^ *in situ*. This estimate is based on linear derating of in-water measurements (UPM-DT-1000PA, Power Meter, Ohmic Instruments, Easton, MD) assuming typical tissue attenuation of 0.5 dB/cm/MHz.

**Figure 2:**
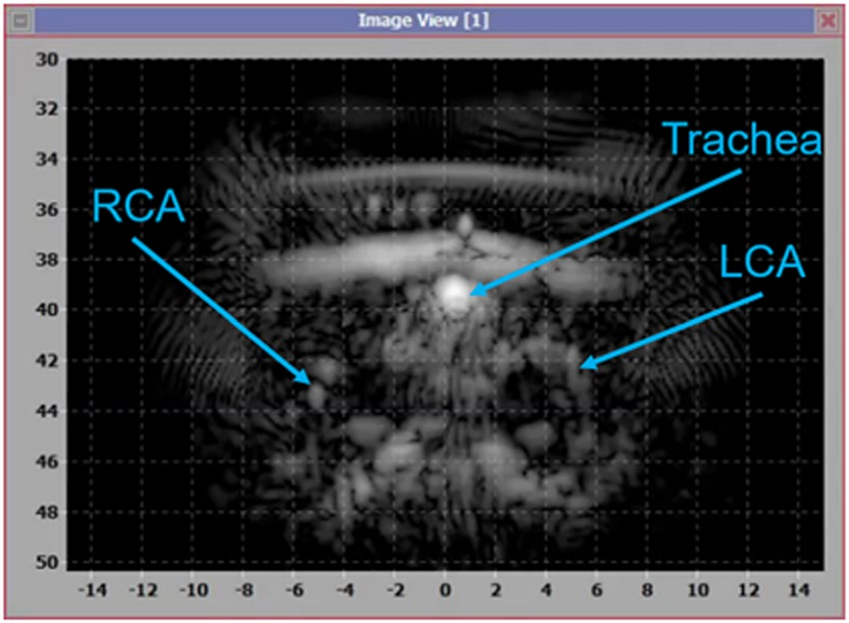
DMUA imaging for identification of the CA bifurcations and other tissue landmarks for guidance.
4. IBS values from the same subblocks were computed and compared to the baseline values. Ratios of IBS for the subblocks were computed in dB with the reference obtained from baseline echoes before the onset of HIFU.
5. If the IBS ratio exceeded a preset threshold (typically 10 dB) for any subblock, HIFU intensity was dropped by 75% after a prespecified hold time (typically 40 ms). The HIFU bursts during this state are referred to as the *sustaining bursts*.
6. HIFU is turned off after reaching the specified HIFU ON time (typically 1 s) or a specified delay after starting the sustaining bursts (typically 400 ms), whichever is earlier.
7. STF imaging continues at the same frame rate for additional post-HIFU time (3–8 s).

The CLC algorithm has other safety features that triggers the cessation of HIFU upon the detection of severe tissue motion or significant tissue motion at the skin interface, etc. However, the simplified version of the algorithm described herein highlights the key features leading to the reliable production of localized, uniform thermal coagulation within the targeted volume. These are:

1. The intensity of the initialization bursts is chosen to be the lowest value suitable to produce tissue boiling within 100 ms with high probability. Based on our preliminary studies, we have established that stopping FUS immediately upon detection of the first boiling event produces minimal or no damage (Supplementary Figure 3). Therefore, the threshold intensity can be estimated *in situ* before starting the actual treatment. Minimizing this value reduces or eliminates the probability of inducing catastrophic events such as vessel perforation.
2. A significant advantage of the initiation of a boiling event is the blocking of the HIFU beam and minimizing any collateral damage distal to the target. The blocking results from an instantaneous impedance mismatch at the boiling location and increased local absorption of the HIFU beam by cavitation bubbles within the focal region.
3. The blocking and increased local absorption allow for the use of sustaining bursts at 25% of the initiation bursts. This has the effect of maintaining the heating to guarantee the uniformity of thermal coagulation (minimum of 200–300 ms required), but doing so without risking excessive exposure thus minimizing the collateral damage proximal to the target.
4. The exquisite synchronization between imaging and therapy is key to the safe application of IgFUS when above-threshold intensities are used. The DMUA system architecture employs strict timing requirements on data acquisition, beamforming, data processing, and HIFU intensity modulation. It is capable of recognizing and responding to adverse events within milliseconds (e.g. by stopping HIFU), which probably exceeds normal human reaction times.

### Volumetric lesion formation within the carotid Bifurcation

After identifying the bifurcation site, two, three or four planes (0.5 mm apart) were targeted for treatment starting at 0.25 mm anterior to the bifurcation either on the internal CA (ICA) or external CA (ECA). Each IgFUS target plane was chosen individually, and three discrete shots were placed such that there was an overlap between all three lesion locations. For each triplet, one shot targeted the ventral wall of the ICA, one shot targeted the dorsal wall of the ECA and one shot was placed in between the two. The procedure was performed either unilaterally or bilaterally in both WKY and SHR rats with survival times between 3–30 days after IgFUS treatment. Some animals were treated bilaterally with up to 2 weeks between unilateral treatments. Two SHR animals received sham treatments, i.e. all procedures were performed, including DMUA imaging, but without delivering therapeutic FUS. Animals were euthanized at different time points after this procedure to assess the damage to the CB using histology.

### Telemetry

Each cage was placed on a receiver (model RPC1) that was connected to a computer via a Data Exchange Matrix (DSI). Data were acquired and analyzed with Ponemah software (DSI). Systolic and diastolic BP, body temperature, and HR data were collected continuously in real-time at 500 Hz over 10 s every 1 min. In addition, continuous monitoring of MAP was performed at 500 Hz for the duration of the IgFUS procedure.

### Data collection and statistical analysis

BP and HR rates of SHR and normotensive animals were monitored daily in real-time over 10 s every minute for 3 days prior to anesthetizing the animals for IgFUS treatment and after the treatment for up to 30-day period using the telemeter (DSI, St Paul, MN) according to manufacturer’s recommendations.

All statistical analyses were performed using MATLAB’s Statistics and Machine Learning Toolbox (Mathworks, Natick, MA). Daily MAP measurements are presented with box plots, which display the summary statistics of median, interquartile range and extremes. The normality of the data was tested with the Kolmogorov-Smirnov test. For comparing the post-treatment MAP measurements between the different groups, one-way ANOVA was performed on the MAP measurements from selected treatment groups. Post-hoc analysis using the Tukey HSD test was applied to the pairwise comparisons. *P* < 0.05 was considered to indicate a statistically significant difference.

### Extraction of the carotid body

Rats were anesthetized using isoflurane (5% induction, 1–2% maintenance) and a midline incision made in the anterior neck to visualize the carotid artery (CA). The common carotid, external and internal carotid arteries were ligated with 3–0 silk suture. Carefully, this section was removed and fixed in 10% formalin for histological analysis.

### Histology

The carotid artery bifurcation with CB and adjacent tissue were then processed to paraffin blocks, sectioned to prepared H&E stained slides, and evaluated by light microscopy. To increase the likelihood of locating the CB, tissues were step sectioned at 25–200 µm intervals. All tissue slides were evaluated by the study pathologist. Photographs were taken for every step of the procedure.

## Results

### Identification of the carotid artery bifurcation using DMUA

**Figure 3:**
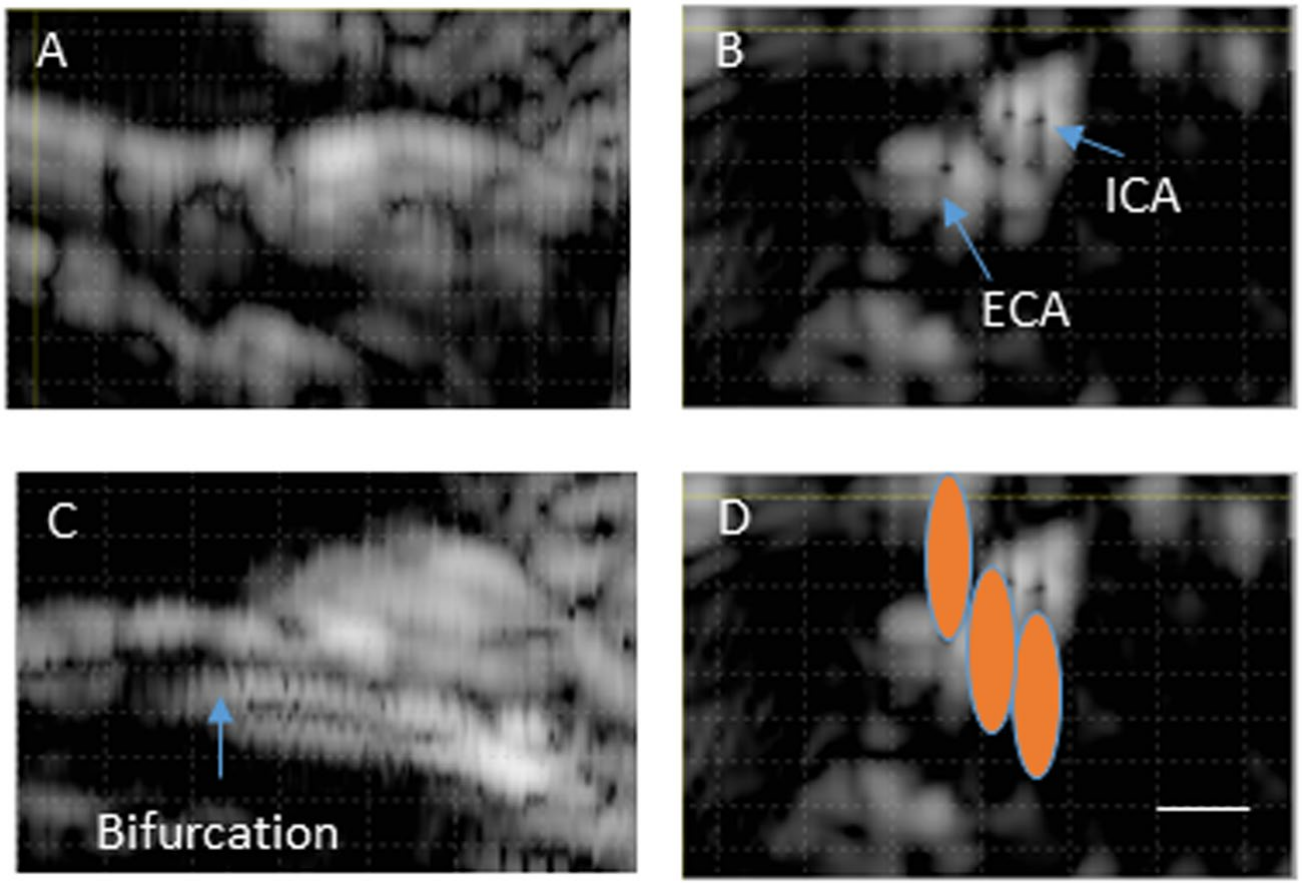
(**A**) B-mode axial view of the right carotid bifurcation of an SHR rat (the main CA is on the left). (**B**) B-mode cross sectional view of the ICA and ECA just cranial to the bifurcation. (**C**) C-scan of the right carotid with arrow pointing to the bifurcation point. (**D**) Schematic representation of the placement of IgFUS shots based on the identification of ICA and ECA in cross-sectional B-mode image.

SHR and normotensive rats were anesthetized and were placed in supine position on the DMUA platform. Hair in the neck area was removed using depilatory cream to provide good coupling for the DMUA transducer. The anatomical region encompassing both the internal CA (ICA) and the external CA (ECA) was visualized by placing the DMUA as shown in Fig. 1. Real-time DMUA imaging allowed for the identification of major anatomical markers such as the trachea, which was used as a reference in the initial placement of the transducer. The carotid arteries produced visible specular reflections and were identified through their pulsation. These specular reflections were most pronounced when imaging the common carotid arteries (CCAs) as shown in Fig. 2. Once the CCA was identified, the DMUA was moved laterally so that the target artery was approximately in the center of the field-of-view (FOV). Three-dimensional image data was collected by acquiring 2D DMUA images while performing a mechanical scan in the posterior-anterior direction (50–100 slices/mm). The bifurcation was identified on rendered orthogonal 2D views of the vessels as shown in Fig. 3. This result is typical of over 80 animal experiments performed using this procedure. On rare occasions, the visibility of the bifurcation was not satisfactorily established and no lesions were attempted.

### Targeted ablation of the carotid bifurcation using DMUA

**Figure 4:**
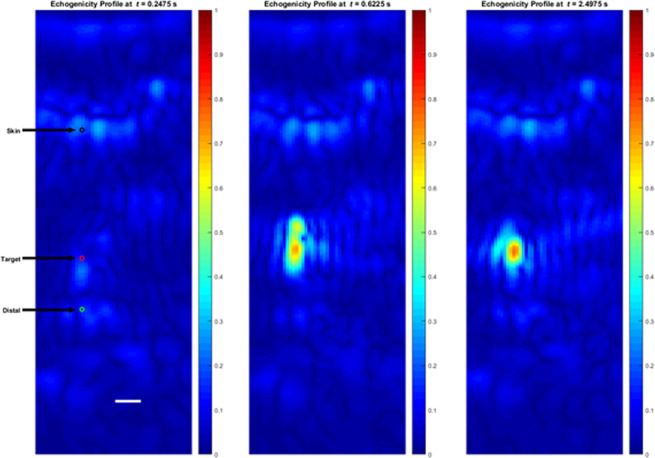
Echogenicity maps from STF frames acquired before (left), during, and after the application of IgFUS. The target (red circle) was the dorsal wall of the ECA. Scale bar represents 1 mm distance.

Figure 4 shows three STF images before (left), during (middle) and after (right) the controlled delivery of a HIFU shot at the dorsal wall of the ECA approximately 1.25 mm anterior to the bifurcation. The target point is indicated by the red circle on the left panel. The vessel wall echogenicity before lesion formation was comparable to that of the skin. During and after lesion formation, the echogenicity of the target location exhibited marked increase and was high enough to trigger the closed-loop control to modulate the intensity as described below. The results shown in Fig. 4 illustrate the spatial extent of the echogenicity change, which correlates well with the dimensions of the focal size of the therapeutic beam (FWHM: 2 mm axially and 400 µm laterally).

**Figure 5:**
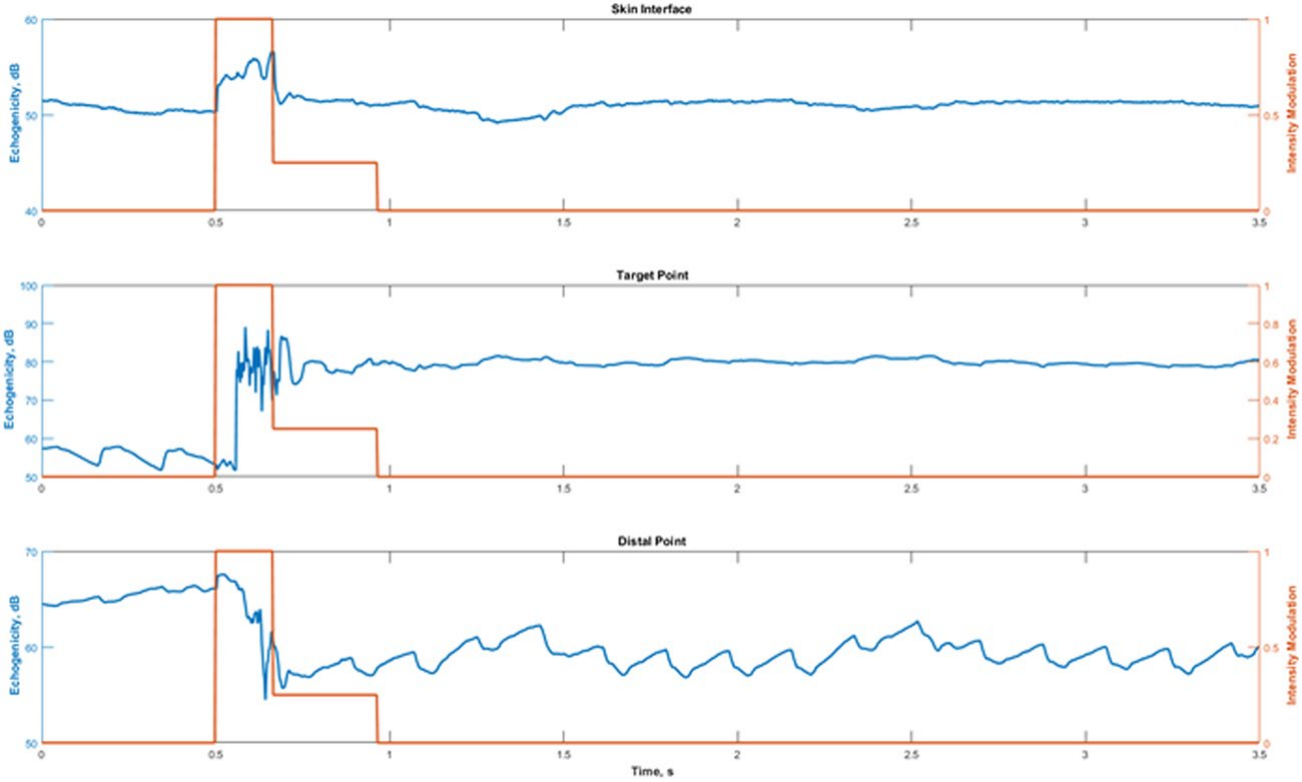
Echogenicity profiles with the CLC intensity modulation profile superimposed. Top: At a point at the skin interface (black circle in Fig. 4). Middle: At the target point. Bottom: At a point 2 mm ventral to the target (green circle in Fig. 4).

The dynamics of the echogenicity change and the CLC intensity modulation are demonstrated by Fig. 5, which shows the temporal echogenicity profiles at the skin (top), target (middle) and a point 2-mm dorsal to the target. The exact locations are indicated by the circles shown on the left panel in Fig. 4. The data was obtained from 1400 STF frames collected over 3.5 s, including 0.5 s (200 frames) of baseline data before the HIFU start time. In each panel, the HIFU intensity modulation profile (red) is superimposed to illustrate the dynamics of the CLC. In this case, the HIFU start time was 0.5 s and the initiation intensity was held for 156.5 milliseconds. The maintenance intensity was held for 300 milliseconds bringing the total HIFU time to 456.5 milliseconds.

The changes in echogenicity at the three different locations highlight the advantages of DMUA imaging with high frame rate data to capture the spatiotemporal evolution of the tissue response to the application of HIFU. For example, at the skin, the echogenicity change was instantaneous due to the effect of radiation pressure at the interface, especially during the initiation phase. The echogenicity change at the target was markedly different, exhibiting a sudden change 56.5 milliseconds after HIFU start and a chaotic behavior for the remainder of the initiation phase. This is a classic tissue boiling event, which was confined to 2-mm spatially as can be seen in Fig. 4 (middle panel). The target point profile also shows the change in echogenicity does not subside immediately after the cessation of HIFU, even though transient changes in the shape continued to occur after the stopping the HIFU application (Fig. 4, right panel).

The distal point profile illustrates the shadowing effect of tissue boiling on the transmission of FUS beyond the event location. In this case, the distal point was located in a high echogenicity region 2-mm ventral to the target (Fig. 4, left panel, green circle). The echogenicity increased immediately after the onset of HIFU, probably due to radiation pressure. However, a sharp drop occurred after the start of the boiling event. This can be explained by the presence of cavitation bubbles within the target region in the path of the STF imaging beam, which resulted in increased reflection and local absorption. Consequently, the incident intensity at the distal point was reduced measurably. This was observed in all cases where a tissue-boiling event was initiated.

### Real-time monitoring of vital signs

**Figure 6:**
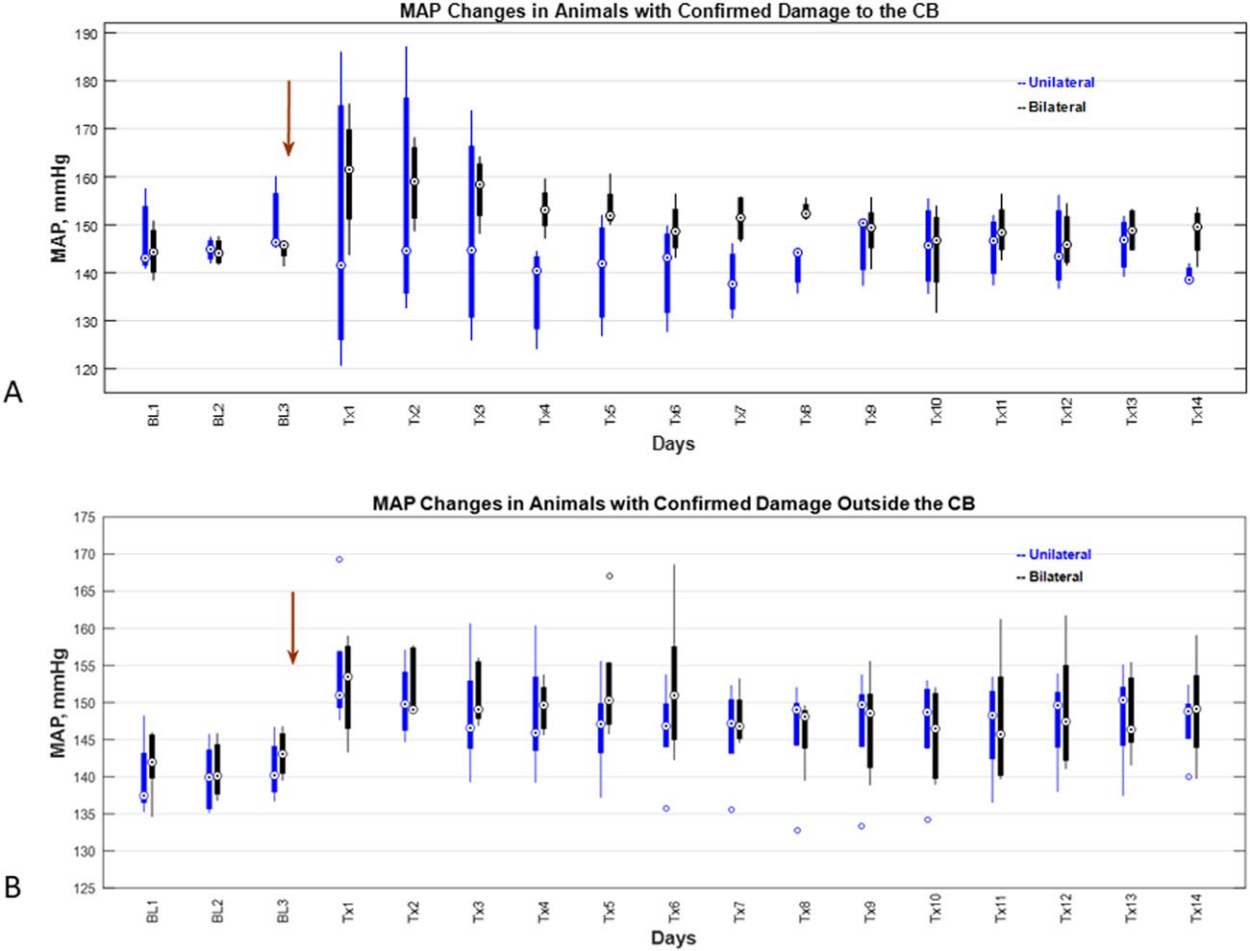
Real-time monitoring of blood pressure in SHR rats that received thermal therapy with DMUA. Mean arterial pressure (MAP) data were plotted against time (in number of days). (**A**) Unilaterally (n = 3) and bilaterally (n = 4) treated animals showed a decrease in the MAP when CB was directly targeted, and (**B**) Unilateral (n = 6) and bilateral (n = 5) treatment with FUS to the regions adjacent to the CB showed an increase in the MAP.

Figure 6A shows MAP measurements from IgFUS-treated SHR rats with confirmed damage to the CB. Immediately after the FUS delivery to the CB, the mean arterial pressure of SHR rats showed an initial spike in bilaterally treated animals. However, it decreased gradually throughout the two-week period tested, but remained higher than the initial baseline level (i.e., MAP present in animals prior to the FUS treatment). On the other hand, unilaterally treated SHR rats showed an initial increase in MAP on day 2 that decreased drastically on day 4 before reaching the baseline on day 10 post-treatment. In general, the overall MAP readings of bilaterally treated rats were significantly higher than in the unilaterally treated SHR animals for 14 days post-treatment. A one-way analysis of variance of post-treatment daily MAP measurements for three groups (unilateral (n = 3), bilateral (n = 4) and sham (n = 2)) showed that the effect of the treatment was statistically significant (F(2, 123) = 8.22, p = 0.0004). Post hoc analyses indicated that the average post-treatment MAP for the unilateral group (M = 144.1, SE = 1.46) was significantly lower than the bilateral (M = 151.7, SE = 1.27) and the sham (M = 150.5, SE = 1.79) (Supplementary Fig. 4A).

Figure 6B shows MAP measurements from IgFUS-treated SHR rats with confirmed damage in the vicinity of the CB, i.e. no significant damage to the CB. In this group, both unilateral and bilateral FUS delivery to the adjacent area just above the CB resulted in a sharp increase in the MAP on day 1 post-treatment, which remained higher than the baseline value for the entire 14-d period tested (Fig. 6B). There was no significant difference in the post-treatment daily MAP measurements between the unilaterally, bilaterally and sham treated animals [F(2,165) = 2.16, p = 0.119 (Supplementary Fig. 4B).

The results shown in Fig. 6A,B suggest that the direct unilateral FUS treatment of the CB might be sufficient to reduce the blood pressure in hypertensive rats. The key to this is the successful targeting of the CB with minimal collateral damage to the surrounding tissues and vessel walls.

### Histological examination of the damaged CB region

**Figure 7:**
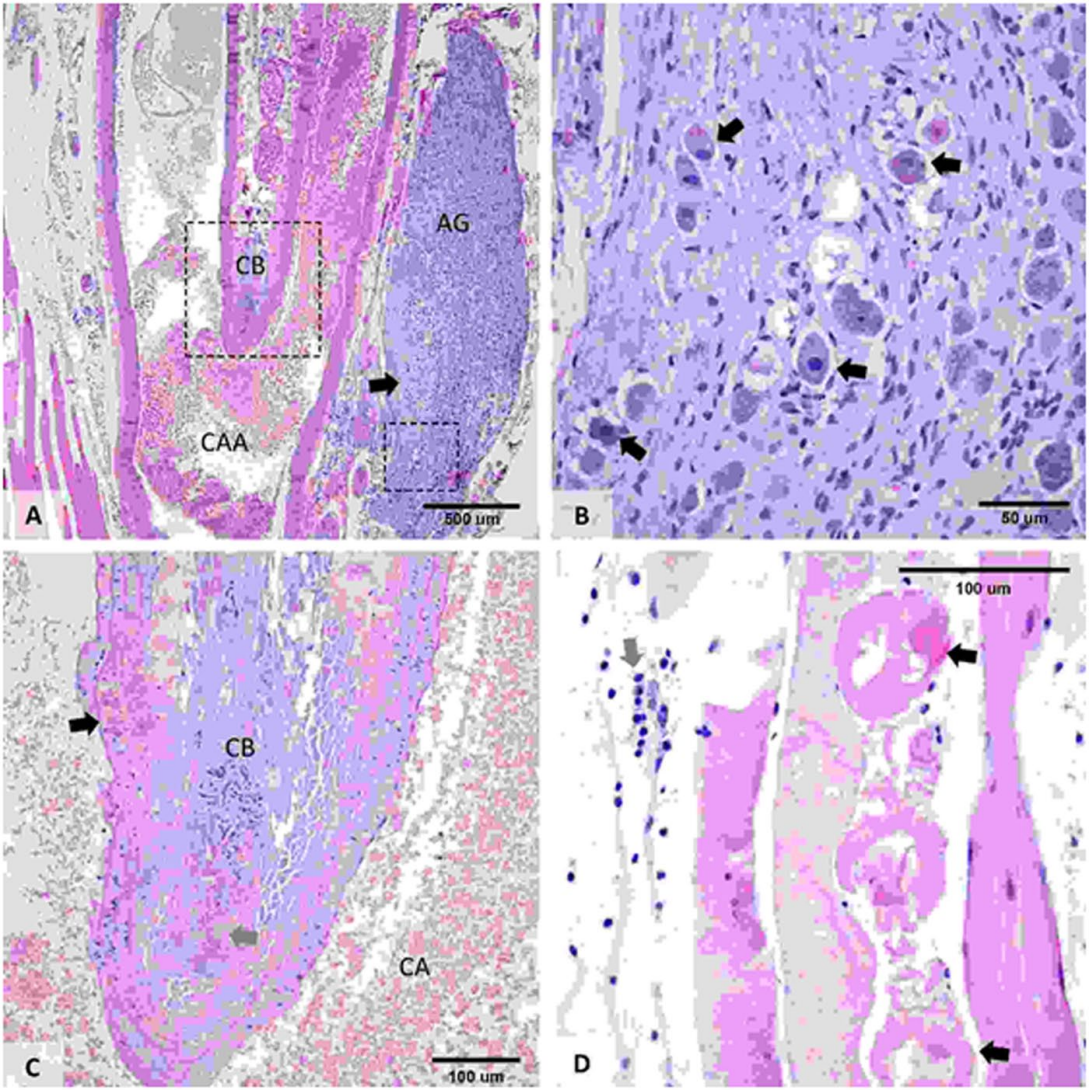
Histopathological feature of carotid body lesions. (**A**) Location of the CB at the bifurcation point of the common carotid artery (CCA) in its internal and external branches. The surrounding tissue is represented by striated muscle (left part of the image) and autonomic ganglion (AG) (embedded in connective tissue). The arrow indicates the focal to extensive coagulative area of necrosis affecting the autonomic ganglion and adjacent tissue. (**B**) Focal to extensive coagulative necrosis was present in AG; and central chromatolysis, perikaryon eosinophilia, vacuolation and karyopyknosis were identified (indicated by the arrows). (**C**) Focal to extensive coagulative necrosis, congestion and hemorrhage (indicated by the grey arrow) that extended to the adjacent walls (indicated by the black arrow). (**D**) Presence of focal rhabdomyolysis (myonecrosis) with loss of visible cross-striations, sarcoplasmic fragmentation, karyopyknosis and karyorrhectic nuclei near the periphery of the CB (indicated by the black arrows). The endomysial space is dilated, edematous, with discrete neutrophil and macrophage infiltration (indicated by the grey arrow).

Histological evaluation of the longitudinal cross-section of the DMUA-treated carotid artery at the bifurcation of the external and internal branches has displayed focal-extensive necrosis with tissue disruption, mild edema, glomus cell eosinophilia with nuclear pyknosis and rhexis (Fig. 7). CB displayed focal to extensive coagulative necrosis, congestion and hemorrhage, and parenchymal disorganization was observed at the periphery of the CB. The region adjacent to the carotid body is largely intact, but slight extension of the tissue damage, such as mild disruption of the elastic lamina, endothelial cell and muscle cell necrosis, and a minimal amount of fibrin at the place of injury, was observed in one case. The region at the periphery of the thermal damage revealed the infiltration of immune cells, primarily neutrophils, leukocytes, and macrophages. However, 2-wk post-DMUA treatment slight regeneration of the CB was evident.

**Figure 8:**
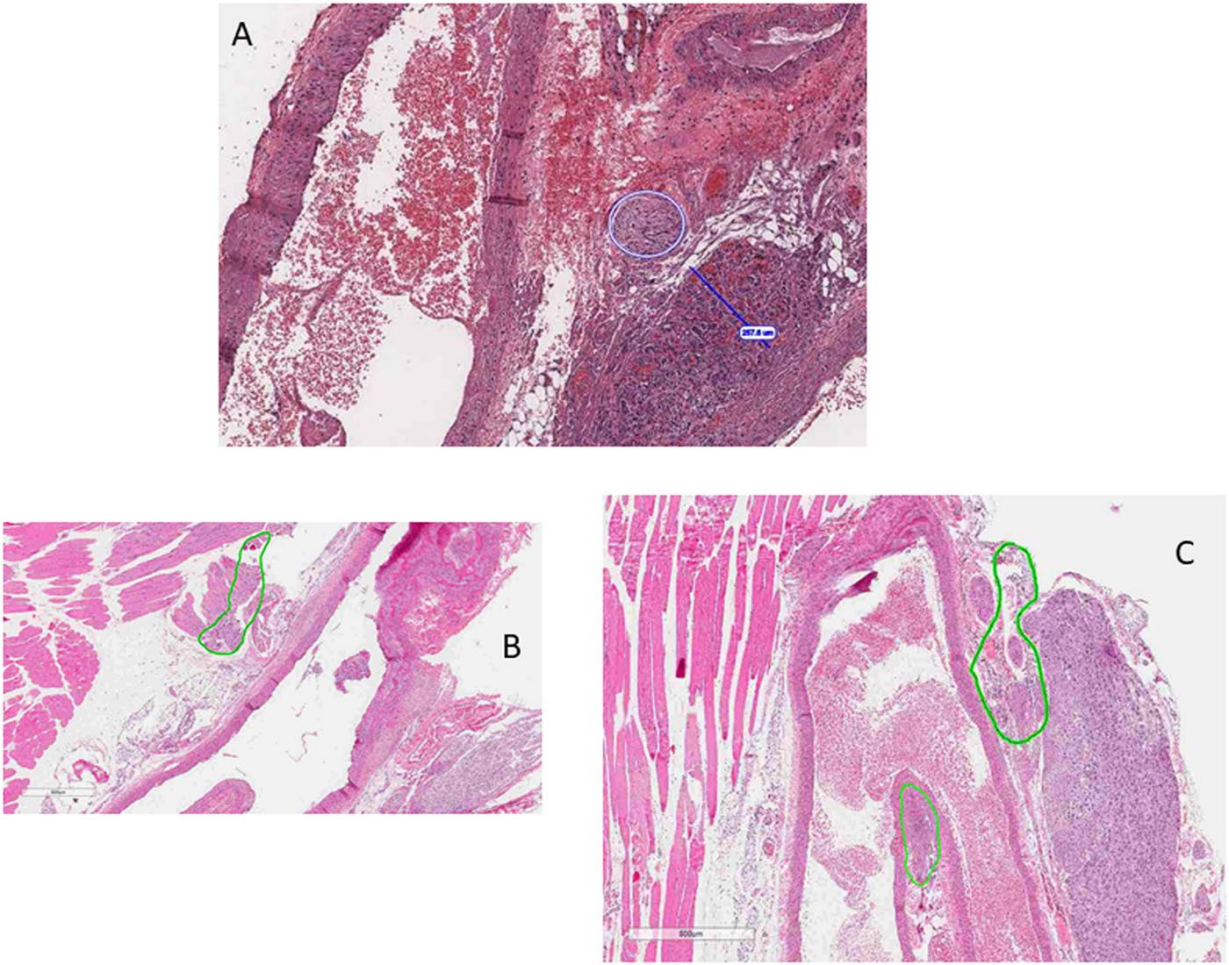
DMUA-induced damage to the carotid body. (**A**) Single shot of FUS delivered using the DMUA system typically induced a lesion of 257 µm. The lesion can be seen either as one (**B**) or two on each side of the blood vessel (**C**).

Animals that received controlled IgFUS treatment directly at the carotid region exhibited extensive necrosis of the CB (Figs. 7 and 8). We also observed marked necrosis of the nerve lining the outer wall of the internal branch of the carotid artery. For these animals, necrosis affected the entire structure of the CB and is characterized by complete loss of cells with remnant stromal architecture, although focal coagulative necrosis is present in the cranial end of the CB. In addition, one animal that was targeted by the DMUA had extensive necrosis of the autonomic ganglion located above the cranial area of the CB and the necrotic process was coagulative and affected virtually all the glomus cells of the CB (Fig. 7). Focal to extensive coagulative necrosis in the autonomic ganglion affecting the adjacent muscle tissue, which was associated with edema and the infiltration of neutrophils, and in the walls of the ECA was found. Focal rhabdomyolysis with loss of visible cross-striations, sarcoplasmic fragmentation, karyopyknosis and karyorrhectic nucleic was also present in the damaged CB region. While IgFUS shots caused severe damage to the CB, in some cases, they also induced damage to the carotid artery at the branching point (at the emergence of both internal and external branches) and expanded further into the external wall of the internal carotid and autonomous ganglion. In other animals, they caused necrosis mainly at the walls of the external carotid artery with mild edema. As shown in Fig. 8, using our CLC approach, we were able to target the CB with a single IgFUS shot that resulted in the lesion of 257.6 µm (Fig. 8A). In general, a single IgFUS shot induced a single lesion that can be seen either entirely on one side of blood vessel (Fig. 8B) or as two lesions that appeared on each side of the vessel (Fig. 8C).

In addition to the above observations, we also noticed a focal area of necrosis affecting the serosa and media of the carotid artery, and a focal rupture of several elastic laminae from intima and media in histological sections that received IgFUS treatment outside the CB region. The defects are partially replaced by fibrous connective tissue, smooth muscle fibers, endothelium and few inflammatory cells. Adjacent to the above described focus, the sympathetic trunk (presumptively), and vagus nerve, present a segmental area of degeneration and necrosis consisting of axonal and Schwann cell vacuolation, swelling, and myelin fragmentation. In a few animals, a small area within the vagus nerve showed mild axonal swelling, myelin fragmentation and minimal cell necrosis with the infiltration of leukocytes. However, the area of damage to the sympathetic trunk and vagus nerve is mild, where the connective tissue adjacent to these structures was diffusely infiltrated by macrophages, lymphocytes and eosinophils.

## Discussion

Thermal bioeffects of focused ultrasound have been investigated for decades. They have the potential to provide a noninvasive alternative to surgical resection^20,47,48^. However, progress has been relatively slow due lack of image-guidance and control. This especially true when FUS is used to target heterogeneous tissues undergoing motion and deformation such as the carotid bifurcation. The results presented herein demonstrate the feasibility of reliable formation of thermal lesions that are confined within the small target volume. Furthermore, collateral damage to the nearby vessel walls was minimal. This was achieved by closed-loop control of IgFUS intensity to: (1) initiate localized tissue boiling at the target within 100 ms of the application of IgFUS beam at intensity levels above the threshold. The initiation of such event increases the therapeutic efficacy and safety by increasing local absorption and blocking further transmission of the beam to more distal tissue, and (2) modulate the intensity to guarantee the formation of uniform thermal coagulation within the target volume. It should be emphasized that high frame rate DMUA imaging feedback and real-time application of the CLC are the key to the safety of the approach. DMUA imaging provides the highest levels of sensitivity and specificity to FUS-induced tissue modifications due to the inherent registration between the imaging and therapeutic coordinate systems.

Previous studies have employed ultrasound-based methods as imaging techniques to visualize CBs or to detect and diagnose CB tumors in animals and in patients with hypertension, sleep apnea, and CB tumors^2,49,50^. In this report, we were able to identify the carotid bifurcation in the rat model, and ablate the CB using a DMUA system to localize the bifurcation as an anatomical marker. Our results demonstrate that the noninvasive ablation of carotid bodies in animals with this system is a safe method as rats that received treatments have tolerated the procedure well. Furthermore, the use of real-time CLC intensity modulation minimized the applied FUS energy. Extensive histological examination of HIFU-induced damage within and around the CB demonstrated the feasibility of this approach as a potential alternative to resection^7^. Targeting the CBs requires accurate imaging of the entire bifurcation region. DMUA imaging was solely used to identify the CA bifurcation as the main anatomical marker for targeting the CB. While these results allowed us to calibrate the FUS exposure levels necessary to produce uniform thermal coagulation within the target volume, future studies will emphasize the direct targeting of the CB itself. It is worth noting recent reports demonstrating the feasibility of identifying the CB in human patients using Doppler ultrasound^2^.

In this study, we treated the rats bilaterally based on a report that the unilateral carotid sinus denervation (CSD; either on the left or right side of the animal) showed no change in the BP of SHR rats^11^. However, this report demonstrated a decline in the BP level when the bilateral CSD was performed with a gap of 15 days between each CSD. We performed bilateral ablation of CB with a gap of 48–72 h between each thermal treatment. In our study, we also observed a decline in the BP of treated SHR animals which remained steady after a 2-wk period. It did not reach the baseline level of normotensive WKY rats (Fig. 6). One probable reason for this is that the thermal damage did not ablate the entire CB and that the surviving glomus cells are maintaining the high BP levels. Another possibility is the potential regeneration of the CB that is responsible for the higher BP level. Further studies are needed to gain insights into the mechanisms that regulate BP and to alleviate hypertension.

A major consideration for our study has been to maintain thermal exposure levels to minimize direct injury to the adjoining arteries and blood vessels of the CB. Histological evaluation of the thermal damage has revealed lesion formation in the CB with very little damage to the artery walls (Fig. 8). The infiltration of immune cells, including macrophages, neutrophils and leukocytes, into the CB as well as its periphery was observed. The amount of thermal dosage and the exposure time was optimized after observing the lesions in the skeletal muscle close to the bifurcation site and loss of myofibers in our earlier experiments (Supplemental Figs. 1 and 3). Our data shows that the initiation *in situ* intensity of 7.2 kW/cm^2^ reliably produced tissue boiling within 100 ms and associated changes in tissue echogenicity to trigger the CLC intensity modulation. The use of maintenance intensity levels under 2 kW/cm^2^ guaranteed uniform thermal coagulation around the target (~2 mm dorsal-ventral and ~400 µm lateral). DMUA imaging was shown to provide immediate feedback indicative of lesion formation as well as spatial mapping of size and location. All the rats (both SHR and WKY) tolerated this therapeutic treatment well and survived the unilateral procedure.

In this study, we observed a variety of responses in animals that received IgFUS treatment. Lesions in the CB typically consisted of focal extensive necrosis with tissue disruption, congestion, and glomus cell necrosis. In the areas adjacent to the CB, focal coagulative necrosis of the carotid artery with disruption of the elastic lamina, muscle cell necrosis, and arterial wall hypereosinophilia was observed. Endothelial cell necrosis and locally adherent fibrin were also present. In some animals unilateral targeted treatment of the CB resulted in extensive damage to the CB, and caused a decrease in their BP levels (Fig. 6A and Supplementary Fig. 4A). In others, IgFUS treatment above the CB region demonstrated an increase in the BP (Fig. 6B and Supplementary Fig. 4B). This could be due to the damage to the adjacent area slightly above the CB bifurcation that may contain barometric receptors^51,52^ that were potentially activated by the treatment.

Renal sympathetic denervation (RDN) is another approach to reduce BP in patients with resistant-hypertension, and was found to be a safe treatment option in humans^53^. A combination of CSD and RDN was tested in the SHR model of hypertension recently^11^. It was shown that when RDN was followed by CSD or vice versa, a fall in the BP occurred after each surgery, and it remained low throughout the 28-d period of the study. However, reflex bradycardia and pressor responses to sodium cyanide were abolished only after the CSD suggesting that the CSD is a response indicator with an independent mechanism^11^. Additional studies are needed to assess the interactions of arterial pressure after CSD and RDN combination or each procedure alone, and the IgFUS approach described herein is a promising tool to induce both carotid and renal ablations in the same animal. Site-specific DMUA designs would be used for optimizing the focusing gain and imaging quality for RDN and CSD.

In conclusion, the present study demonstrates the feasibility and safety of using IgFUS under DMUA imaging guidance to identify the target carotid bifurcation and perform closed-loop control of lesion formation. Using this approach, lesions were shown to exhibit uniform thermal coagulation within the target tissue with minimal damage to the nearby vessel walls. Histological evaluation confirmed the thermal lesion characteristics and confinement of the therapeutic effects. In cases where damage to the CB was confirmed histologically, we observed measureable reduction of the BP up to 2 weeks after IgFUS treatments. In cases where lesions were formed anterior to the CB (Supplementary Fig. 5), sustained increase in BP was observed for up to 4 weeks. In all cases, lesion boundaries were well defined and damage to the vessel walls was minimal. Combined with the results from our previously reported vascular targeting studies in large animals, the results from the present study warrant the investigation of the CLC approach in large animals and eventually in human patients. It is worth mentioning that the approach described herein is already approved for use in human patients with PVD (atherosclerosis in the femoral artery).

## Supplementary information

Supplementary Table and Figures.

## Data Availability

The datasets generated during and/or analyzed during the current study are available from the corresponding author on reasonable request.
