# Supplementary material for: Precision Targeted Ablation of Fine Neurovascular Structures In Vivo Using Dual-mode Ultrasound Arrays
Source: Sci Rep. 2020 Jun 8;10:9249. doi: 10.1038/s41598-020-66209-0 (PMC7280193; doi:10.1038/s41598-020-66209-0)
Supplement: Supplementary file 1 — Supplementary Table and Figures. [file 41598_2020_66209_MOESM1_ESM.pdf]

Rajagopal N. Aravalli[1], Dusty Van Helden[2], Dalong Liu[1], Parker O'Brien[1], Hasan Aldiabat[1,#], Alexandru-Flaviu Tăbăran[3,#], M. Gerard O'Sullivan[3], H. Brent Clark[4], John W. Osborn[2], and Emad S. Ebbini[1],*

Precision targeted ablation of fine neurovascular structures *in vivo* using dual-mode ultrasound arrays.

Supplementary Table 1. The number of animals and genotype used in this study and their HIFU treatment conditions.

Genotype	Treatment	Number of animals
Wild-type (normotensive)	Unilateral	7
Wild-type	Bilateral	7
Wild-type	Sham	6
SHR	Unilateral	12
SHR	Bilateral	24
SHR	Sham	8

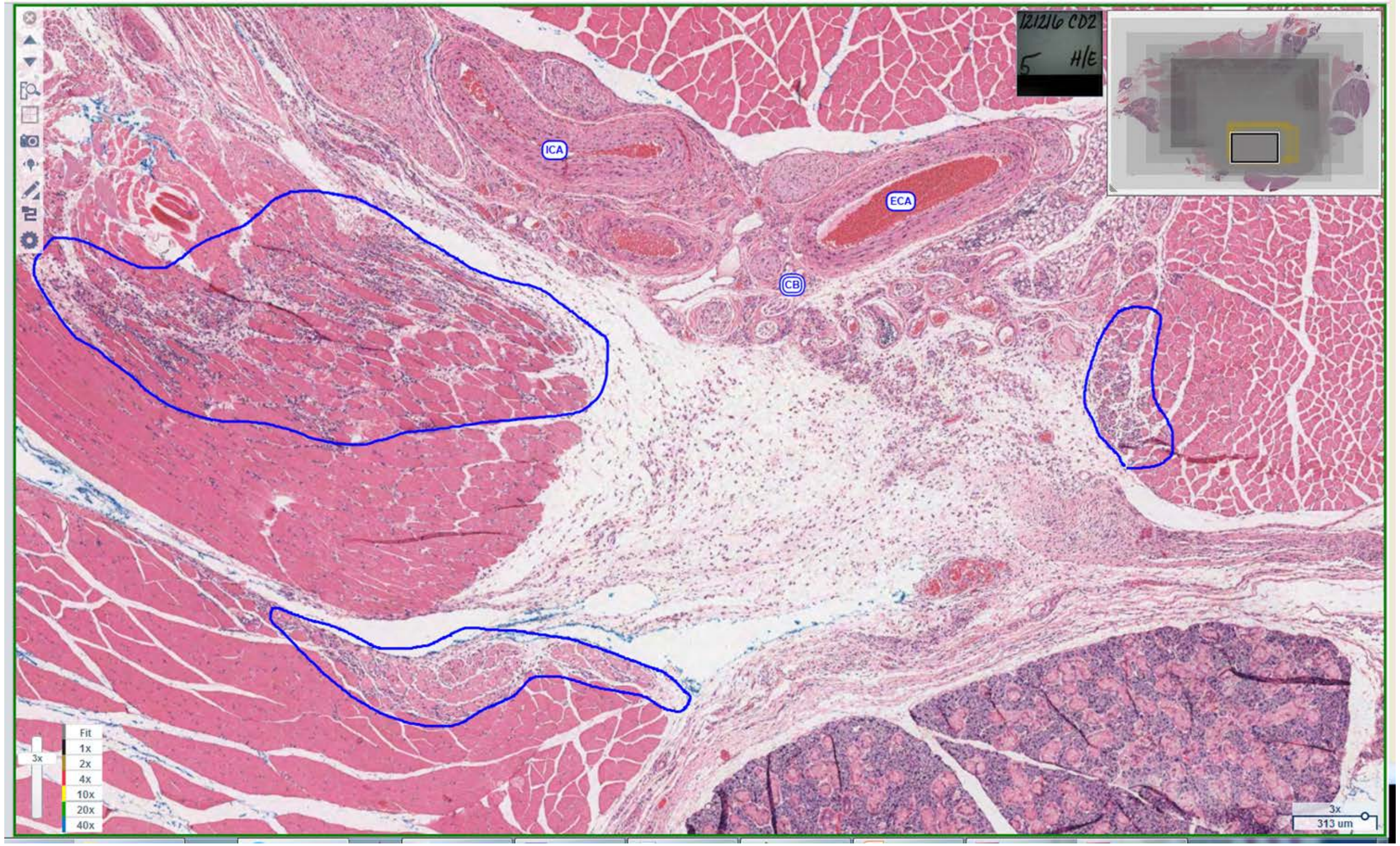

Supplementary Figure 1. Open-Loop Control vs Closed-Loop Control: Larger lesion was formed using open-loop control (9 kW/cm^2 for 1 sec at 50% duty cycle) while the smaller two where formed using CLC with the same initial intensity. The vessels were easily identified on DMUA imaging, but were not directly targeted. Lesions formed using real-time CLC are smaller in the axial direction. In the context of CB targeting, the open-loop control lesions are examples of *overexposure.*

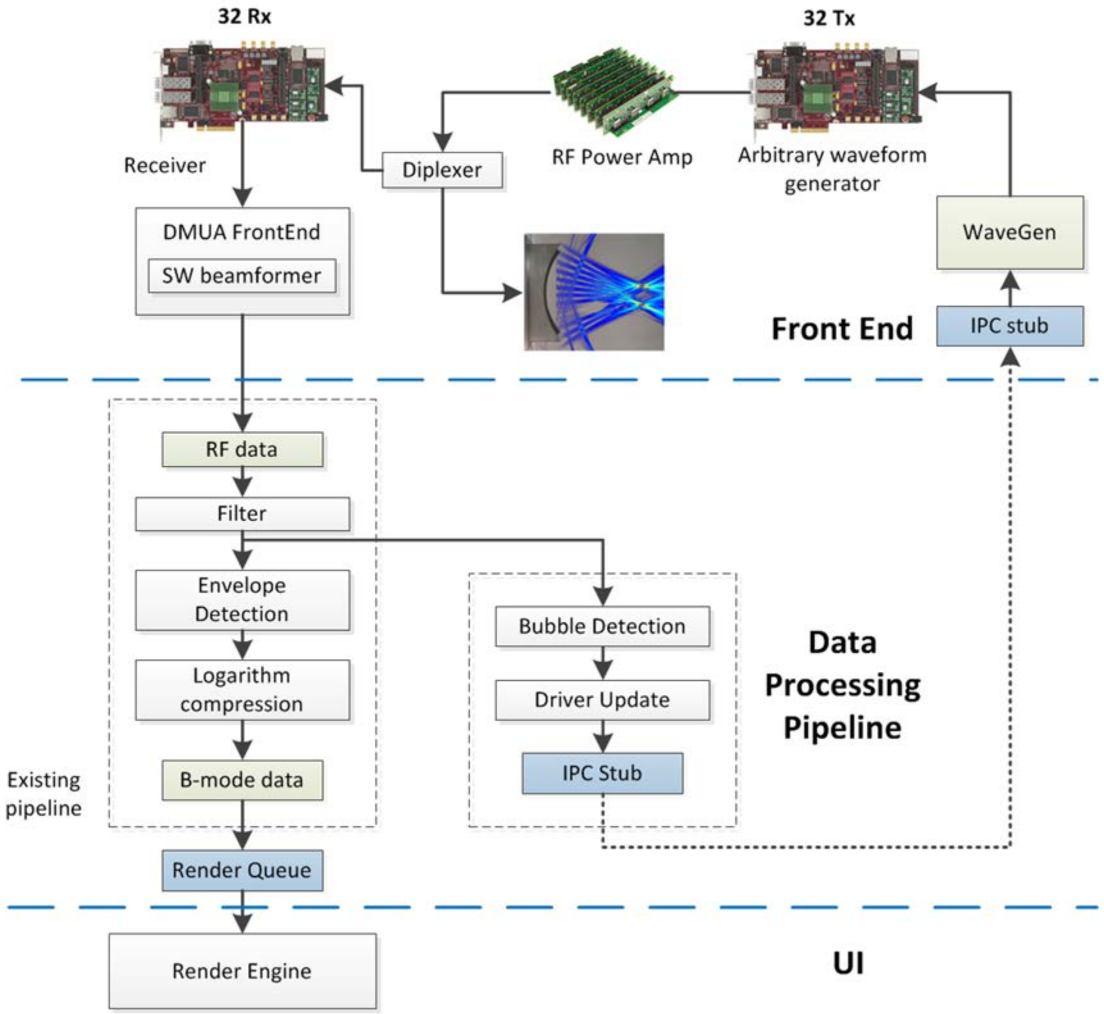

Supplementary Figure 2. Hardware/Software architecture for implementing the real-time CLC lesion formation using STF feedback data.

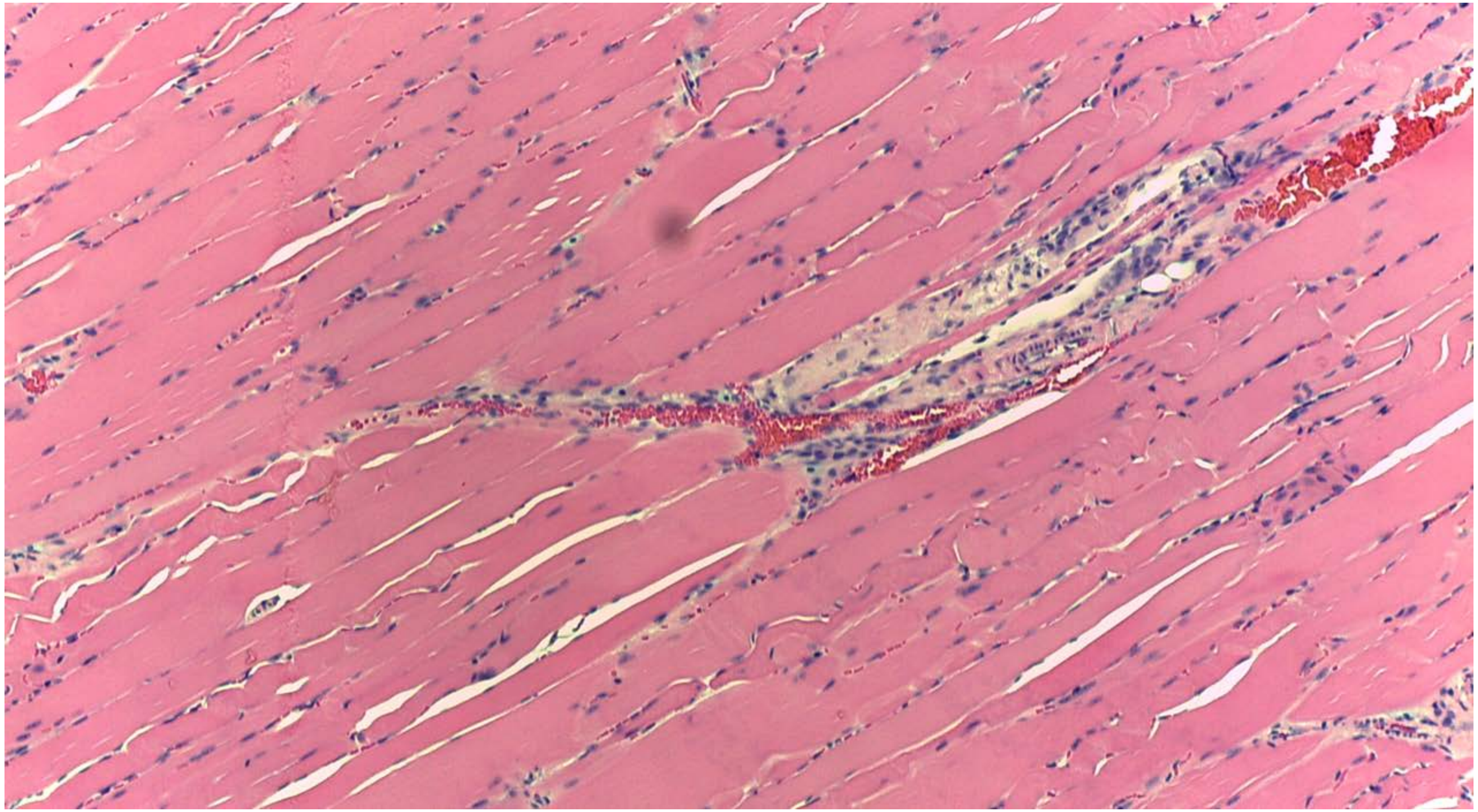

Supplementary Figure 3. Example of *"micro lesion"* formed by using above-threshold initial intensity, but stopping HIFU immediately upon bubble detection. One can count the number of cells affected by this exposure. We have observed healing and regeneration in even larger lesions than the one shown here.

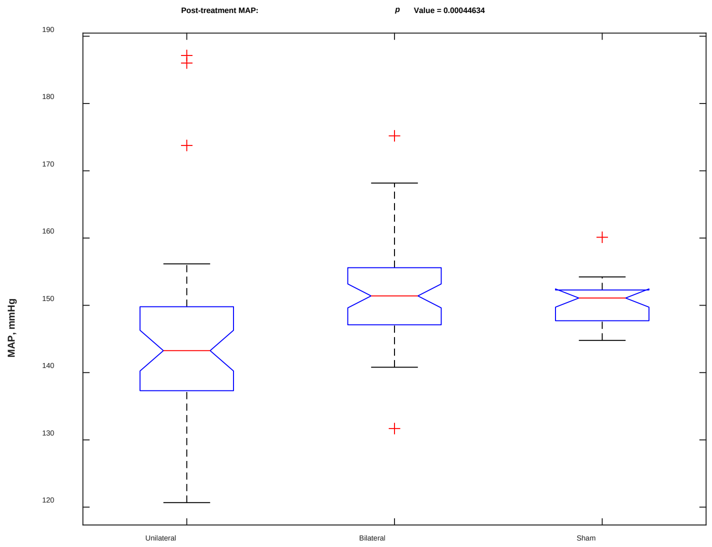

ANOVA Table

Source	SS	df	MS	F	Prob>F
Groups	1480.7	2	740.371	8.22	0.0004
Error	11079.7	123	90.079		
Total	12560.4	125			

A

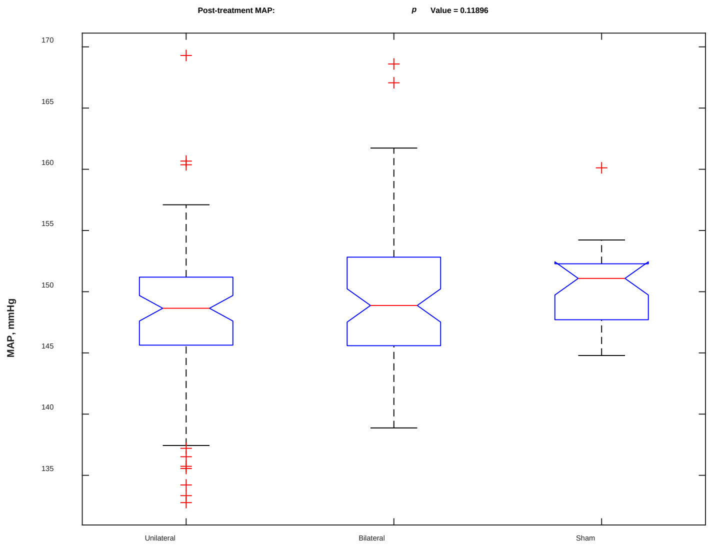

ANOVA Table

Source	SS	df	MS	F	Prob>F
Groups	160	2	79.998	2.16	0.119
Error	6120.3	165	37.0928		
Total	6280.3	167			

B

Supplementary Figure 4. One-way ANOVA of MAP measurements post IgFUS treatment. A) In animals with confirmed damage to the CB (and sham treatment). The analysis is performed on MAP measurements on Tx1 – Tx14 (Fig. 6A in the manuscript). B) In animals where no significant damage to the CB was observed (and sham treatment). The analysis was performed on MAP measurements Tx1 – Tx14 (Fig. 6B in the manuscript). The + marks are outliers.

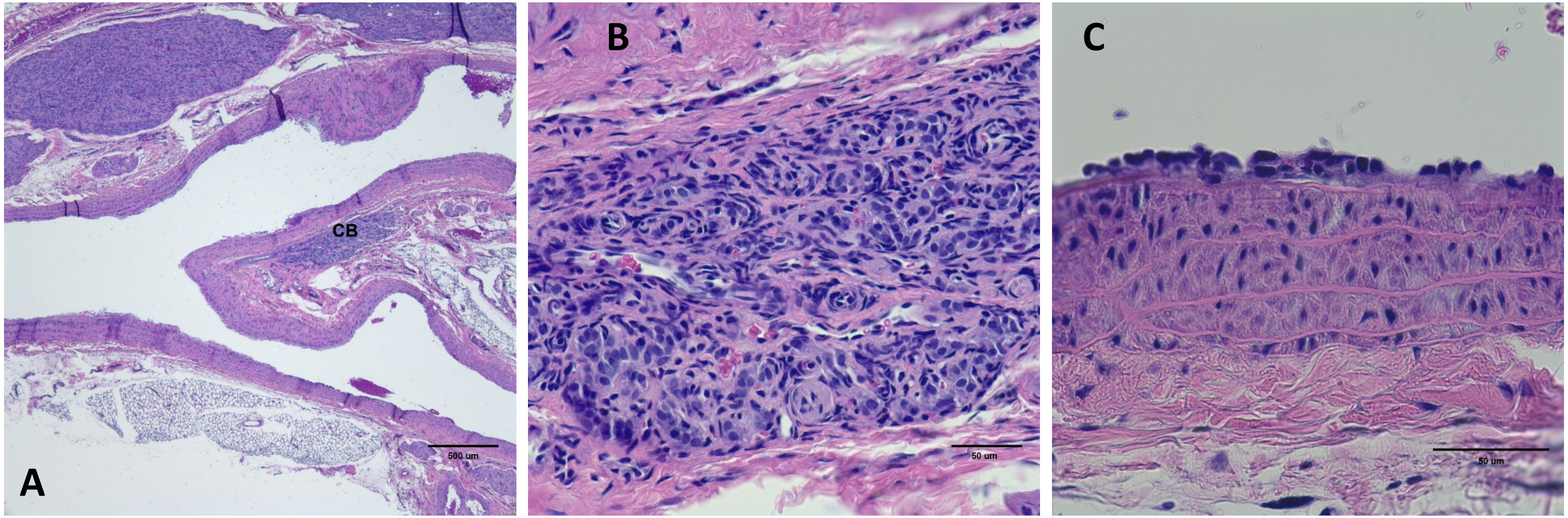

Supplementary Figure 5. MAP increase in animals that received FUS, either unilaterally or bilaterally, in the region immediately adjacent to the carotid body. Area surrounding the CB at the bifurcation of the carotid artery and adjacent tissues were not damaged (A), and CB is intact (B). However, focal areas of endothelial hyperplasia in the external branch of the carotid artery were present (C) . Scale bar: A: 500 µm; B and C: 50 µm.
